# The assessment in patients with acute fatty liver of pregnancy (AFLP) treated with plasma exchange: a cohort study of 298 patients

**DOI:** 10.1186/s12884-023-05503-x

**Published:** 2023-03-13

**Authors:** Lingxia Li, Dengchao Huang, Jing Xu, Miaojing Li, Juan Zhao, Qindong Shi, Qinyue Guo

**Affiliations:** 1 Department of Obstetrics and Gynecology, Xijing Hospital, Air Force Medical University, Xi’an, 710032 China; 2 Department of Critical Care Medicine, The First Affiliated Hospital of Xi’an Jiaotong University, 277 Yanta West Street, Xi’an, 710061 Shaanxi China; 3 Department of Emergency Medicine, The First Affiliated Hospital of Xi’an Jiaotong University, 277 Yanta West Street, Xi’an, 710061 Shaanxi China; 4 Department of Hematopathology, The First Affiliated Hospital of Xi’an Jiaotong University, 277 Yanta West Street, Xi’an, 710061 Shaanxi China

**Keywords:** Acute fatty liver of pregnancy, Plasma exchange, Propensity score match

## Abstract

**Background:**

To assess the prevalence, risk factors, clinical characteristics of Acute fatty liver of pregnancy (AFLP) patients, and outcomes of AFLP patients treated with plasma exchange (PE).

**Methods:**

We retrospectively reviewed the AFLP patients admitted to the First Affiliated Hospital of Xi’an Jiaotong University and Xijing Hospital of Air Force Medical University from January 2012 to May 2022. Final prediction model for death among AFLP by means of stepwise backward elimination with *p* value < 0.05. Patients treated with and without PE were compared by propensity-matched cohort study.

**Results:**

Two hundred ninety eight patients with the diagnosis of AFLP, and finally 290 patients were enrolled in the cohort study, 50 of whom (17.2%) were dead. Compared with AFLP patients alive, the dead of patients were more likely to be combined encephalopathy (*p* < 0.01), postpartum hemorrhage (*p* < 0.01), and found significantly higher frequency of fetal distress (*p* = 0.04), fetal death (*p* < 0.01). we developed a predicted probability value and with an area under the receiver operating characteristics (ROC) curve of 0.94 (95%CI 0.87 to 1.00), indicating AFLP patients’ death. The patients treated with PE had a significantly lower 60-day mortality rate (OR 0.42, 95% CI 0.29 to 2.64, *p* = 0.04), and significantly shorter duration of hospital-free days at day 28 (*p* = 0.01).

**Conclusions:**

In conclusion, our study indicated that liver function were risk factors for maternal mortality, and PE was a protective factor for maternal 60-day mortality and hospital-free days at day 28 in AFLP patients.

## Background

Acute fatty liver of pregnancy (AFLP) is considered to be an obstetrics emergency that presents maternal liver dysfunction and/or failure even multiorgan failure [1], which can lead to maternal and fetal complications, including coma or death [2, 3].

The disease is rare, with incidence values of 1:7000–15,000 pregnancies that typically occurs during the third trimester of pregnancy [4–6]. The potential risk factors for AFLP include Fetal long-chain 3-hydroxyacyl CoA dehydrogenase deficiency; prior episode of AFLP; multiple gestation; preeclampsia or hemolysis, elevated liver enzymes, and a low platelet count (HELLP syndrome), etc. [7]. Although the prognosis has improved during these years, the mortality remains high, which maternal mortality has been reported to be 2–12% [6, 8, 9] and perinatal mortality 10–15% [10].

As we known, early diagnosis and early initial management such as timely delivery were very important treatment strategies for AFLP patients cause of fulminant liver failure may not be reversible, study reported that if they were delivered within a week as soon as the disease diagnosed, 100% cases could survive, while 30% cases would die if they were delivered beyond 2 weeks after onset [11]. In addition to early delivery, supportive treatment are also very important part of the management of AFLP, such as critical care support for patients and fetus, monitoring for and treatment of hypoglycemia, coagulopathy, mechanical ventilation for acute respiratory distress syndrome (ARDS), N-acetylcysteine treatment, dialysis, or plasmapheresis [12, 13], beyond these, liver transplantation has been explored as a last measure, but its use remains controversial [14].

Unfortunately, there are still no series or larger observational studies to provide evidence for these supportive approaches above, more research are needed to deepen understanding and optimize management of AFLP. We performed this retrospective cohort study to investigate the risk factors, clinical manifestations, and outcomes, further evaluate the therapeutic effect of plasmapheresis management among patients with AFLP patients.

## Methods

### Study population

We retrospectively reviewed the medical records of all adult patients with confirmed AFLP who were admitted to the First Affiliated Hospital of Xi’an Jiaotong University and Xijing Hospital of Air Force Medical University from January 2012 to May 2022. The diagnosis of AFLP was based on the compatible clinical manifestation and laboratory findings, and all patients met the Swansea criteria [1]. The patients with ①chronic hepatitis B, chronic hepatitis C and liver cirrhosis, ②accidental death, such as pulmonary embolism, ③abandon treatment were excluded. This study was approved by the institutional review board. Informed consent was waived due to the retrospective nature of the study.

### Study design

**Fig. 1:**
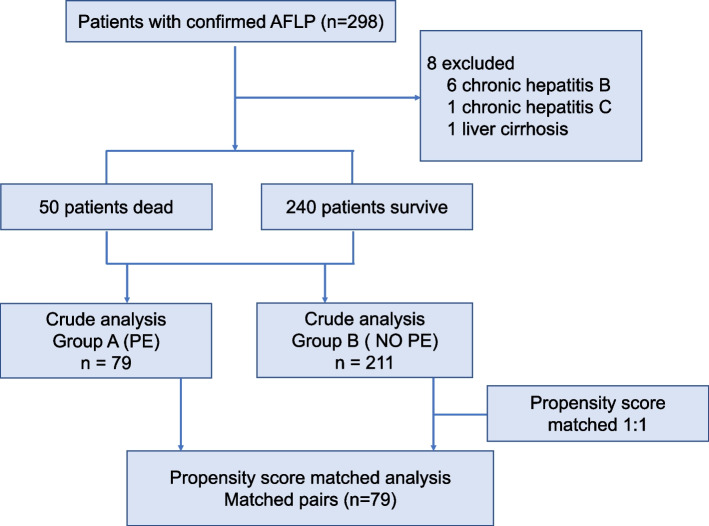
Schematic flow chart for a cohort study

The first part of this study was a retrospective cohort study that included all patients with confirmed AFLP, we planned to predict the risk factors for death in AFLP patients. The second part of the study was a retrospective cohort study, we divided 290 patients into two subgroups, group A represented patients treated with plasma exchange (PE) and group B represented patients treated without PE, the outcome of patients was compared among these two groups. A schematic flow chart is shown in Fig. 1.

### PE implementation

The decision to undertake PE in AFLP patients were decided by the supervising physician team (obstetricians and critical care intensivists, gastroenterologist) based on the severity of the illness, which according to the current therapeutic indications [15]. Informed consent singed by the family once decided to initiate PE. Fresh frozen plasma as the dominant replacement fluid with a total plasma volume target 8-12L or 1–1.5L daily until disease control.

### Definitions

The Swansea criteria is commonly used to diagnose AFLP, including vomiting, abdominal pain, polydipsia/ polyuria, encephalopathy, bilirubin > 0.8 mg/dl, hypoglycemia < 4 mmol/l, leukocytosis > 11*10^3^/mL, elevated transaminases (AST or ALT) (> 42 international unit/L), elevated ammonia (> 47 μmol/L), elevated urate (> 340 μmol/L), acute kidney injury, or creatinine > 150 μmol/L, coagulopathy (prothrombin time (PT) > 14 s, imaging: Ascites or bright liver on ultrasound scan, histology: microvesicular steatosis on liver biopsy. Six or more of these terms are required to diagnose AFLP [8, 16, 17]. Encephalopathy means a syndrome of overall brain dysfunction, in our study, mainly included hepatic encephalopathy, hypoxic ischemic encephalopathy, and Wernicke’s encephalopathy [18]. Infections were caused by infectious pathogens including bacteria, viruses, and fungi. Other Complications including intestinal obstruction, intestinal adhesion, hepatorenal syndrome, hemorrhage of digestive tract and acute respiratory distress syndrome (ARDS). The differential diagnosis of AFLP involves the HELLP syndrome, severe symptoms and signs of hepatic insufficiency such as hypoglycemia, encephalopathy and coagulopathy, multi-organ involvement, especially renal failure are more common with AFLP than HELLP, while hypertension and proteinuria are often more severe in HELLP.

### Data collection

Data collected included: (1) demographics (Maternal age at first AFLP pregnancy, body mass index [BMI], sex, age, and living habits); (2) Pregnancy information: gravidity, multiparae, fetal sex male, gestational age, multiplets; (3) comorbidities were collected during or after delivery: such as hypertension before pregnancy, preeclampsia, hypothyroidism during pregnancy, gestational diabetes, encephalopathy, infection, postpartum hemorrhage, HELLP syndrome, and referred to fetal diseases (placenta previa, placental abruption, fetal distress, fetal death); (4) the course of AFLP: date of onset, symptoms at presentation, date of hospital admission, and parturition; (5) laboratory findings on first visit: complete blood count, blood chemistry, coagulation indicator, and infectious biomarkers (white blood cell [WBC]); (6) treatment: plasma exchange, bilirubin adsorption, continuous renal replacement therapy (CRRT) and cesarean section, hysterectomy.

### Statistical analysis

Measurement data were expressed by means of descriptive statistics, including mean ± standard deviation (SD) and 95% CI. Categorical variables were examined by Fisher exact test or chi-square test, as appropriate, while continuous variables were compared by student t test or Mann–Whitney U test. Logistic regression was used to analyze relative factors. All tests of significance were two-tailed and *P* < 0.05 was considered statistically significant.

In order to account for potential confounding factors in this observational study, we developed a propensity score, using multivariate logistic regression analysis without regards to outcomes [19, 20], to adjust for the differences in baseline characteristics between AFLP patients with and without PE. All prespecified covariates were included in the final prediction model for death among AFLP patients, by means of stepwise backward elimination with *p* value < 0.05. Model discrimination was assessed by area under the receiver operating characteristics curve (AUROC). The statistical approach was similar to our previous study [21].

The effect of AP on 60-day mortality, as well as hospital-free days at day 28 were analyzed by stepwise backward logistic regression model by including any covariate with *p* value < 0.10 in univariate analysis. In addition, we performed a nested case–control study (1:1 match) by matching case and control subjects, a nearest-neighbor matching algorithm was employed to form pairs of case and control subjects. Survival curves for case and control subjects were analyzed by the Kaplan–Meier method and compared by log-rank test. Results were analyzed with SPSS version 23.0 K for Windows (SPSS Inc., Chicago, IL, USA) and GraphPad Prism 9 (GraphPad Software, San Diego, CA, USA).

## Results

### Patient enrolment and clinical characteristics

During the study period, 298 patients with the diagnosis of AFLP, and 8 patients were excluded due to chronic hepatitis B, chronic hepatitis C and liver cirrhosis, 290 patients were included in the final analysis, the patients’ disposition was shown in Fig. 1.

The clinical characteristics of patients were shown in Table 1, of totally two hundreds and ninety cases, including fifty-seven were multiparae (*n* = 57, 19.7%), two hundreds and thirty-three were male of fetal sex (*n* = 233, 80.3%), and 8.6% were multiplets (*n* = 25, 8.6%). The women were 27.21 ± 4.95 years of age at their AFLP diagnosis, gave birth at a mean gestational age of 253.10 ± 17.58 days. The majority comorbidities were fetal distress (*n* = 130, 44.82%), encephalopathy (*n* = 81, 27.93%), infection (*n* = 60, 20.68%), fetal death (*n* = 78, 26.89%). Cesarean section and CRRT were used in 267 (92.1%) and 107 (36.9%) patients, respectively. A total of 79 patients (27.24%) received Plasma exchange (PE) treatment, and 22 patients (7.59%) received bilirubin adsorption treatment. 34 patients took in the surgery of hysterectomy (11.72%).

**Table 1:** Stratification analysis between the risk factors and death of AFLP patients

	TOTAL (*n* = 290)	SURVIVAL (*n* = 240)	DEATH (*n* = 50)	*P* value
Maternal age at first AFLP pregnancy, y, (mean ± SD)	27.21 ± 4.95	26.71 ± 4.59	29.6 ± 6.43	0.30
BMI, Kg/m^2^, (mean ± SD)	24.07 ± 3.27	24.63 ± 2.90	21.4 ± 3.98	0.58
Gravidity, (mean ± SD)	1.69 ± 0.81	1.67 ± 0.82	1.80 ± 0.84	0.91
Multiparae	57(19.7%)	48(20.0%)	9(18.0%)	0.85
Fetal sex male	233(80.3%)	192(80.0%)	41(82.0%)	0.85
Gestational age, d, (mean ± SD)	253.10 ± 17.58	253.29 ± 17.55	252.20 ± 19.75	0.60
Multiplets	25(8.6%)	23(9.6%)	2(4.0%)	0.27
Days from symptom onset to hospitalization, d, (mean ± SD)	12.89 ± 16.51	8.26 ± 5.14	35.00 ± 32.40	< 0.01
Days from symptom onset to parturition, d (mean ± SD)	14.25 ± 17.20	9.05 ± 4.96	38.20 ± 31.85	< 0.01
APACHE II score, (mean ± SD)	19.61 ± 9.20	12.77 ± 4.96	30.20 ± 3.49	0.03
Swansea score, (mean ± SD)	7.52 ± 1.48	7.38 ± 1.56	8.20 ± 0.84	0.22
**Complications**				
Hypertension before pregnancy	7(2.4%)	5(2.1%)	2(4.0%)	0.42
Preeclampsia	54(18.6%)	42(17.5%)	12(24.0%)	0.32
Hypothyroidism during pregnancy	6(2.1%)	4(1.7%)	2(4.0%)	0.28
Gestational diabetes	17(6.6%)	14(5.8%)	3(6.0%)	0.99
Encephalopathy	81(27.9%)	43(17.9%)	38(76.0%)	0.01
Infection	60(20.7%)	48(20.0%)	12(24.0%)	0.57
Postpartum Hemorrhage	39(13.4%)	26(10.8%)	13(26.0%)	0.01
HELLP syndrome	34(11.7%)	25(10.4%)	9(18.0%)	0.15
Placenta previa	33(11.4%)	28(11.7%)	5(10.0%)	0.99
Placental Abruption	39(13.4%)	33(13.8%)	6(12.0%)	0.99
Fetal distress	130(44.8%)	101(42.1%)	29(58.0%)	0.04
Fetal death	78(26.9%)	53(22.1%)	25(50.0%)	< 0.01
Others	19(6.6%)	12(5.0%)	7(14.0%)	0.03
**Lab on first visit (mean ± SD)**				
Hemoglobin(g/L)	112.00 ± 25.24	115.58 ± 25.60	94.80 ± 15.64	0.29
Platelet (*10^9^/L)	141.66 ± 80.49	133.33 ± 78.48	181.60 ± 86.78	0.95
TBil (mmol/L)	154.70 ± 99.52	146.54 ± 89.28	193.86 ± 145.56	0.02
DBil (mmol/L)	132.13 ± 94.21	122.83 ± 81.29	176.74 ± 145.35	0.17
ALT (IU/L)	314.59 ± 658.66	116.20 ± 35.10	355.92 ± 719.44	0.03
PT (s)	22.49 ± 19.67	21.73 ± 21.38	26.14 ± 7.79	0.75
APTT(s)	49.23 ± 16.76	45.70 ± 14.41	66.18 ± 18.40	0.33
Prothrombin activity (%)	48.74 ± 18.20	52.83 ± 16.54	29.12 ± 12.28	0.06
Scr (umol/L)	190.76 ± 76.41	187.67 ± 73.01	205.6 ± 99.40	0.27
BUN (mmol/L)	7.47 ± 3.88	7.80 ± 4.05	5.90 ± 2.61	0.41
WBC(*10^6^/L)	15.81 ± 6.16	16.40 ± 6.18	12.96 ± 5.82	0.54
Trioxypurine (μmol/L)	360.62 ± 148.87	351.13 ± 149.08	406.20 ± 155.59	0.59
**Management**				
Plasma exchange	79(27.2%)	68(28.3%)	11(22.0%)	0.39
Bilirubin adsorption	22(7.6%)	10(4.2%)	12(24.0%)	< 0.01
CRRT	107(36.9%)	82(34.2%)	25(50.0%)	0.04
Cesarean section	267(92.1%)	223(92.9%)	43(86.0%)	0.15
Hysterectomy	34(11.7%)	32(13.3%)	2(4.0%)	0.09

Data are presented as the number (percentage) of patients unless indicated otherwise

*AFLP* Acute fatty liver of pregnancy, *BMI* Body Mass Index, *APACHEII score* Acute Physiology and Chronic Health Evaluation, *TBil* Total bilirubin, *DBil* Direct bilirubin, *ALT* Alanine transaminase, *PT* Prothrombin time, *APTT* Activated partial thromboplastin time, *Scr* Serum creatinine, *BUN* Blood urea nitrogen, *WBC* White blood cells, *CRRT* Renal replacement therapy, *ICU* Intensive care unit

### Clinical characteristics and managements of AFLP patients associated to death

In 290 patients with AFLP in this study, survival group contained 240 patients (82.8%), and a total of 50 patients (17.2%) were dead. As shown in Table 1, the death group had longer days from symptom onset to hospitalization and days from symptom onset to parturition than the survival group (*p* < 0.01, respectively), and APACHE II score in death group were higher (*p* < 0.05). Compared with AFLP patients alive, the dead of patients were more likely to be combined encephalopathy (*p* < 0.01), postpartum hemorrhage (*p* < 0.01), and other complications (*p* = 0.03). In terms of fetal/infant complications, a significantly higher frequency of fetal distress (*p* = 0.04), fetal death (*p* < 0.01) was found in death group than in survival group. There was no significant difference between dead and survival patients regards to laboratory examination and pregnancy information (gravidity, multiparae, fetal sex male, gestational age, and multiplets) except the total bilirubin (TBil) and alanine transaminase (ALT) (*p* < 0.05, respectively) (Table 1).

In addition, patients in death group were more likely to receive treatments of Bilirubin adsorption and CRRT, these differences had statistical significance (*p* < 0.01, respectively). Interestingly, there was no significant difference between the two groups with regards to management of plasma exchange, cesarean section, and hysterectomy (Table 1).

**Fig. 2:**
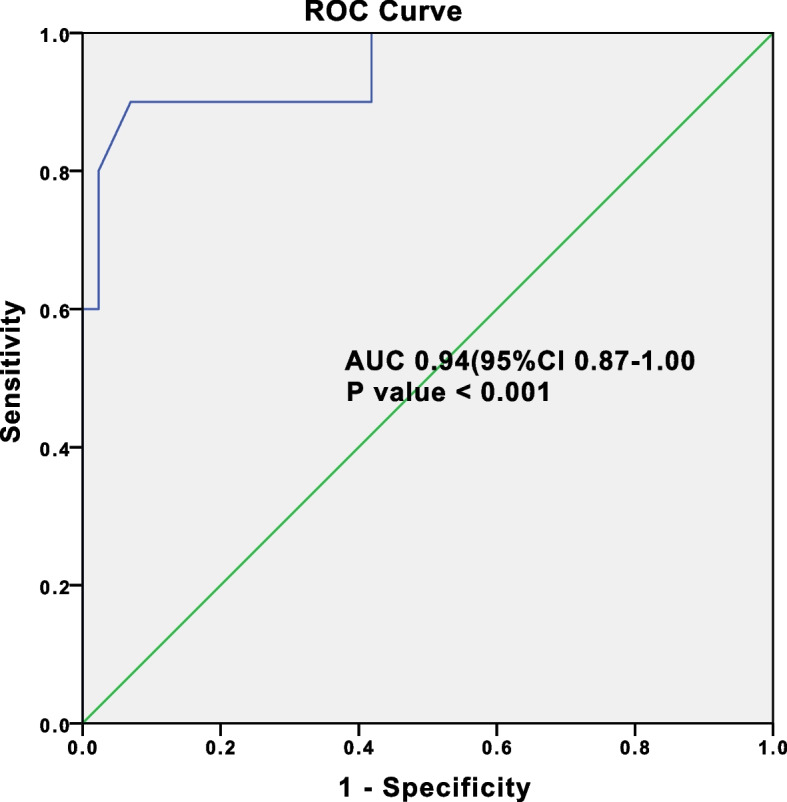
Receiver operating characteristics (ROC) curve for a AFLP patient’s likelihood of death

Based on above data which *p* value < 0.05 (including days from symptom to hospitalization and parturition, the APACHE II score; complications as encephalopathy, postpartum Hemorrhage, and fetal illness such as fetal distress, fetal death; the high TBil and AST levels of meternity; the management such as Bilirubin adsorption, plasma exchange, and CRRT), we developed a predicted probability value and using a binary logistic regression model with an area under the receiver operating characteristics curve of 0.94 (95%CI 0.87 to 1.00) (Fig. 2), indicating good discrimination of AFLP patients’ death.

### Patients with PE and development of propensity score

The clinical characteristics of the AFLP patients received PE treatment or not were summarized in Table 2, a total of 79 patients (27.2%) were treated with PE (Table 2). As is show in Table 2, there were a significantly higher or lower frequency of maternal information and obstetrical complications in PE group (group A) than group B: multiparae (10.13% vs. 23.22%, *p* = 0.01), fetal sex male (68.35% vs. 84.83%, *p* < 0.01), multiplets (24.05% vs.2.84%, *p* < 0.01), HELLP syndrome (20.25% vs.8.53%, *p* = 0.01), and placenta previa (21.52% vs. 7.58%, *p* < 0.01). No difference was found of gravidity (1.71 ± 0.76 vs. 1.68 ± 0.84, *p* = 0.72), gestational age (241.43 ± 16.56 vs. 256.82 ± 16.54, *p* = 0.78), APACHEII score (*p* = 0.78) and Swansea score (*p* = 0.71) between two groups (Table 2).

**Table 2:** Baseline characteristics of AFLP patients received PE or not

	GROUP A PE (*n* = 79)	GROUP B NO PE (*n* = 211)	*P* value	^a^*P* value
Maternal age at first AFLP pregnancy, y, (mean ± SD)	26.86 ± 3.63	27.32 ± 5.37	0.38	0.11
BMI, Kg/m^2^, (mean ± SD)	23.71 ± 3.69	24.18 ± 3.22	0.50	0.89
Gravidity, (mean ± SD)	1.71 ± 0.76	1.68 ± 0.84	0.72	0.69
Multiparae	8(10.13%)	49(23.22%)	**0.01**	0.50
Fetal sex male	54(68.35%)	179(84.83%)	** < 0.01**	0.72
﻿Gestational age, d, (mean ± SD)	241.43 ± 16.56	256.82 ± 16.54	0.78	0.10
Multiplets	19(24.05%)	6(2.84%)	** < 0.01**	0.77
Days from symptom onset to hospitalization, d, (mean ± SD)	9.80 ± 7.05	14.89 ± 20.00	0.41	0.55
Days from symptom onset to parturition, d, (mean ± SD)	10.80 ± 6.22	16.26 ± 20.53	0.30	0.57
APACHEII score, (mean ± SD)	19.75 ± 10.87	17.00 ± 9.04	0.97	0.16
Swansea score, (mean ± SD)	7.86 ± 1.35	7.41 ± 1.53	0.71	0.07
**Complications**				
Hypertension before pregnancy	2(2.53%)	5(2.37%)	0.99	0.99
Preeclampsia	15(18.99%)	39(18.48%)	0.99	0.54
Hypothyroidism during pregnancy	1(1.27%)	5(2.84%)	0.68	0.32
Gestational diabetes	5(6.33%)	12(5.69%)	0.79	0.66
Encephalopathy	22(27.85%)	59(27.96%)	0.99	0.27
Infection	11(13.92%)	49(23.22%)	0.10	0.25
Postpartum Hemorrhage	8(10.13%)	31(14.69%)	0.34	0.89
HELLP syndrome	16(20.25%)	18(8.53%)	**0.01**	0.67
Placenta previa	17(21.52%)	16(7.58%)	** < 0.01**	0.56
Placental Abruption	11(13.92%)	28(13.27%)	0.85	0.76
Fetal distress	33(41.77%)	97(45.97%)	0.60	0.50
Fetal death	22(27.85%)	56(26.54%)	0.88	0.72
Others	3(3.80%)	16(7.58%)	0.30	0.45
**Lab on first visit (mean ± SD)**				
Hemoglobin(g/L)	112.43 ± 26.66	111.86 ± 25.43	0.62	0.17
Platelet (*10^9^/L)	145.71 ± 81.71	140.36 ± 81.99	0.99	0.09
TBil (mmol/L)	202.03 ± 107.10	139.64 ± 94.59	0.86	0.88
DBil (mmol/L)	175.16 ± 95.80	118.44 ± 91.64	0.81	0.88
ALT (IU/L)	160.86 ± 158.22	363.50 ± 748.94	0.37	0.45
PT (s)	34.57 ± 38.01	18.65 ± 6.25	** < 0.01**	0.39
APTT(s)	53.77 ± 15.23	47.78 ± 17.30	0.68	0.22
Prothrombin activity (%)	41.93 ± 10.06	50.91 ± 19.80	**0.07**	0.64
Scr (umol/L)	225.14 ± 39.38	179.82 ± 82.59	**0.052**	0.68
BUN (mmol/L)	8.77 ± 5.82	7.06 ± 3.01	**0.07**	0.08
WBC(*10^6^/L)	17.45 ± 9.11	15.28 ± 5.07	** < 0.01**	0.10
Trioxypurine (μmol/L)	391.14 ± 144.48	350.91 ± 152.23	0.63	0.69
**Management**				
Bilirubin adsorption	20(25.32%)	2(0.95%)	** < 0.01**	NA
CRRT	66(83.54%)	41(19.43%)	** < 0.01**	NA
Cesarean section	77(97.47%)	190(90.05%)	**0.04**	0.53
Hysterectomy	13(16.5%)	21(9.9%)	0.43	0.52

Data are presented as the number (percentage) of patients unless indicated otherwise

*AFLP* Acute fatty liver of pregnancy, *PE* Plasma exchange, *BMI* Body Mass Index, *APACHEII score* Acute Physiology and Chronic Health Evaluation, *TBil* Total bilirubin, *DBil* Direct bilirubin, *ALT* Alanine transaminase, *PT* Prothrombin time, *APTT* Activated partial thromboplastin time, *Scr* Serum creatinine, *BUN* Blood urea nitrogen, *WBC* White blood cells, *CRRT* Renal replacement therapy

^a^Of 290 patients, 79 pairs were matched

When compared the laboratory examination we found that higher mean values of prothrombin time (PT) (34.57 ± 38.01 vs. 18.65 ± 6.25, *p* = 0.01) and white blood cells (17.45 ± 9.11 vs. 15.28 ± 5.07, *p* = 0.003) in Group A. Besides, more patients in Group A received bilirubin adsorption (25.32% vs.0.96%, *p* < 0.01), CRRT (83.54% vs.19.43%, *p* < 0.01) and cesarean Sect. (97.47% vs.90.05%, *p* = 0.04) other than hysterectomy (*p* = 0.44) (Table 2).

In propensity score-matched cohort study, 79 AFLP patients treated with PE were successfully matched with 79 AFLP patients without PE (Table 2), including 46 cases for exact matches and 33 cases for fuzzy matches. There were no significant differences of the baseline characteristics between the matched patients with and without PE.

### Plasma exchange as a protective factor for clinical outcome

**Fig. 3:**
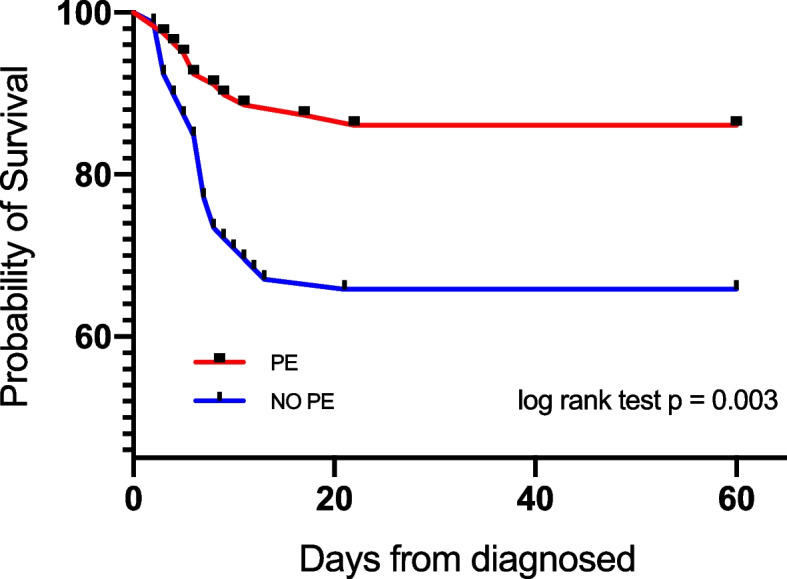
Survival curves stratified by all AFLP patients with PE management were compared with 79 matched subjects who without

There was no significant difference of maternal crude 60-day mortality rates (*p* = 0.65) and hospital-free days at day 28 (*p* = 0.85) between Group A and Group B (Table 3). After propensity score matching, PE group had a significantly lower 60-day mortality rate (OR 0.42, 95% CI 0.29 to 2.64, *p* = 0.04) (Table 3 and Fig. 3), and shorter hospital-free days at day 28 (SD 13.57, 95% CI 4.02 to 20.11, *p* = 0.01) (Table 3).

**Table 3:** Comparison of PE treatment associated with outcomes in AFLP patients

	Crude		Matched^a^	
**Clinical Outcome**	OR (95% CI)	*P* value	OR (95% CI)	^a^*P* value
60-day mortality	0.75(0.70–8.02)	0.65	0.42(0.29–2.64)	0.04
	Std.Error Difference (95% CI)		Std.Error Difference (95% CI)	
Hospital-free days at day 28, d (mean ± SD)	4.08(-10.03–7.4)	0.85	4.38(4.02–20.11)	0.01

*PE* Plasma exchange, *AFLP* Acute fatty liver of pregnancy, *CI* Confidence interval, *OR* Odds ratio

^a^Of 290 patients, 79 pairs were matched

## Discussion

As we mentioned above, AFLP is a rare but potentially fatal disease [8]. In these years, with the increased awareness, timely diagnosis and delivery of the fetus and improvements in obstetric intensive care, the maternal all-cause mortality rates for AFLP have improved, which was drop down to around 20% worldwide, which from close to 100% [3, 4, 22]. Nevertheless, AFLP remains the most dangerous disease to both mother and fetus [23]. Early recognition and diagnosis are very important for AFLP, in addition, timely termination of pregnancy and supportive care were key points for improve survival rate [24, 25]. So, for better understand the disease, more studies are needed for suppling its clinical characteristics, treatments, and outcomes [26].

The association between AFLP and potential risk factors were well documented by the public literature [4–7], which was not much different from our results, our study showed most cases were fetal sex male, but the occurrences which easy to cause case had found not associated with death outcomes in patients with AFLP, such as male fetal sex, multiple gestation, preeclampsia, nulliparity, we also found the death patients seems to had lower body mass index (BMI) (21.4 ± 3.98 vs. 24.63 ± 2.90) but there was no statistical significance.

It was unquestioned that initial management of the AFLP patient with includes prompt delivery of the fetus, regardless of gestational age, because delivery initiates resolution of this life-threating disease [27]. As we shown, the days from symptom onset to hospitalization and parturition were the high-risk factors for AFLP death, so we suggested that once the underlying symptoms are identified for pregnant women, especially the symptoms which are easily to ignored, such as fatigue and cold food preference [17, 28], or once a patient is diagnosed with AFLP, plans should be made to proceed with prompt delivery. Additionally, earlier diagnosis for outpatient pregnant women could be life-saving [29, 30].

The severity of illness on the first day admission was expressed by APACHE II score, obviously significantly higher in death group, it is different from Swansea criteria which is justified for the diagnosis of AFLP, but it is not targeted at predicted death. Complications and laboratory data are the key tools in diagnosing AFLP and distinguishing from other more common liver diseases in pregnancy such as preeclampsia, HELLP syndrome, and intrahepatic cholestasis of pregnancy [8, 31]. Encephalopathy was an important characteristic which can distinguish AFLP from diseases above, the occurrence of complications is also associated with maternal death outcomes [31, 32], our study verified that encephalopathy, postpartum hemorrhage are the most significant and life-threatening maternal complications and fetal distress and fetal death for neonatal complications. Besides, TBil and ALT were risk factors for maternal mortality. Combination of all the significantly conditions shown a high risk of death, early recognition of patients with these potentially life-threatening conditions might be very important to improve clinical outcome.

Another major finding of our study was that PE was a protective factor for 60-day mortality and hospital-free days at day 28 in AFLP patients, which had rarely verified in the current studies. As we know, plasma exchange (PE) as adjuvant therapy which using automated machinery to simultaneously remove a desired volume of a patient’s plasma and replace it primarily with pooled fresh frozen plasma, has been successfully used as adjuvant therapy for pregnant patients such as severe preeclampsia and HELLP syndrome [26, 33]. The temporizing effect of PE is thought to occur by the removal of circulating endotoxins, replacement of normal coagulation factors and proteins, interruption of coagulopathy, and finally by improving renal function [34, 35]. However, PE as a main support system designed to treat patients with liver failure fall concluded that had no significant effect on mortality compared with standard medical therapy in a systematic review that pooled 12 randomized controlled trials [36, 37].

Our results were consistent with Gao’s previous study [38], which demonstrated that total bilirubin was independent risk factors for maternal mortality. Thus, whether the therapy of plasma exchange could improve the survival rate of AFLP patients? In 1989, the first case reported that PE was used for AFLP patient [26], this two decades, there was increasing experience with treatment of PE for AFLP patients, but rarely reported, the statistical data on survival rates due to treatments effects of PE were rarely reported. Our results supplemented the data that verify PE treatment is really beneficial to AFLP patients, specifically, the significantly lower mortality rate and shorter hospital-free days at day 28. So, we suggested PE should use as an important treatment choice for AFLP patients with hyperbilirubinemia.

The reason why crude 60-day mortality and hospital-free days at day 28 had no significant differences was its presence a particularly high-risk group of patients for whom treated with PE. The major strength of our study was the robustness of the study result, which was supported by univariate analysis, multivariate regression analysis adjusted for propensity score, and 1:1 propensity score-matched cohort study [39, 40], the main advantage of this approach is minimize the bias, finally obtain the reliable conclusions, which was the same as our previous research [21]. Our study was also subject to limitations. First, the specific details need to be refined, such as the data of timing and the running days of PE had not covered in this report, which need to be analyzed in further study, may help us to determine the best time of PE. Second, it is a common belief that plasma exchange is safe and effective for patients with AFLP, so we did not pay close attention to the side effects of PE [41]. Last, it does limit the generalizability of the results that there was an overrepresentation of women from the Asia, but the results still useful and meaningful.

## Conclusions

In conclusion, our study indicated that liver function were risk factors for maternal mortality, and PE was a protective factor for maternal 60-day mortality and hospital-free days at day 28 in AFLP patients.

## Data Availability

The datasets analyzed during the current study are available from the corresponding author upon reasonable request.

## Abbreviations

AFLP: Acute fatty liver of pregnancy
PE: Plasma exchange
HELLP syndrome: Hemolysis, Elevated liver test, Low platelet count
ARDS: Acute respiratory distress syndrome
ALT: Alanine aminotransferase
AST: Aspartate aminotransferase
PT: Prothrombin time
WBC: White Blood Cells
CRRT: Continuous renal replacement therapy
BMI: Body mass index
AUROC: Receiver operating characteristics curve
TBil: Total bilirubin
